# Feedforward consequences of isometric contractions: effort and ventilation

**DOI:** 10.14814/phy2.12882

**Published:** 2016-08-01

**Authors:** Billy L. Luu, Janette L. Smith, Peter G. Martin, Rachel A. McBain, Janet L. Taylor, Jane E. Butler

**Affiliations:** ^1^ Neuroscience Research AustraliaRandwickNSWAustralia; ^2^ School of Medical SciencesUniversity of New South WalesSydneyNSWAustralia; ^3^ National Drug and Alcohol Research CentreUniversity of New South WalesSydneyNSWAustralia

**Keywords:** Bilateral, central command, force, human, hypercapnia, muscle

## Abstract

The onset of voluntary muscle contractions causes rapid increases in ventilation and is accompanied by a sensation of effort. Both the ventilatory response and perception of effort are proportional to contraction intensity, but these behaviors have been generalized from contractions of a single muscle group. Our aim was to determine how these relationships are affected by simultaneous contractions of multiple muscle groups. We examined the ventilatory response and perceived effort of contraction during separate and simultaneous isometric contractions of the contralateral elbow flexors and of an ipsilateral elbow flexor and knee extensor. Subjects made 10‐sec contractions at 25, 50, and 100% of maximum during normocapnia and hypercapnia. For simultaneous contractions, both muscle groups were activated at the same intensities. Ventilation was measured continuously and subjects rated the effort required to produce each contraction. As expected, ventilation and perceived effort increased proportionally with contraction intensity during individual contractions. However, during simultaneous contractions, neither ventilation nor effort reflected the combined muscle output. Rather, the ventilatory response was similar to when contractions were performed separately, and effort ratings showed a small but significant increase for simultaneous contractions. Hypercapnia at rest doubled baseline ventilation, but did not affect the difference in perceived effort between separate and simultaneous contractions. The ventilatory response and the sense of effort at the onset of muscle activity are not related to the total output of the motor pathways, or the working muscles, but arise from cortical regions upstream from the motor cortex.

## Introduction

At the onset of a sustained isometric muscle contraction, there is an abrupt increase in ventilation. For moderate to intense contractions, this usually occurs within the first breath after the onset of muscle activation, followed by a slower rise in ventilation (Dejours 1964; Myhre and Andersen 1971). The neural mechanisms that underlie this rapid increase in ventilation during a sustained muscular effort are not completely understood, but several studies indicate that both central and peripheral signals can act on respiratory neurons to increase the drive to breathe.

The currently held view of a centrally generated ventilatory response to voluntary isometric contractions involves a corollary of the central motor command, that is, a signal generated from within the central nervous system, which projects to respiratory premotoneurones to increase ventilation. As such, the size of the corollary signal, and therefore ventilatory response, would be proportional to the descending central motor drive. This view is based on experiments that manipulate the level of central motor command required to maintain a given muscle tension in both static and dynamic tasks. For example, using muscle tendon vibration to alter the motoneuronal excitability of a statically contracting muscle (Goodwin et al. 1972) or by weakening a muscle with partial curarization during cycling (Asmussen et al. 1965; Galbo et al. 1987). In any case, there is a greater than normal increase in ventilation when more central motor command is required to maintain an absolute muscle tension. Evidence that peripheral signals contribute to the initial ventilatory response to muscle activation stems from the study by McCloskey and Mitchell (1972) who showed that when the central motor command is bypassed with electrical stimulation of spinal ventral roots in cats, ventilation increased at the onset of a tetanic muscle contraction, and this increase in ventilation was abolished by preferential blockade of groups III and IV afferents with local anesthesia. Later studies showed that passive dynamic exercise in humans during sleep (Ishida et al. 1993) and wakefulness (Sato et al. 2004; Bell and Duffin 2006) also produced rapid increases in ventilation.

Even though both central and peripheral signals can generate a ventilatory response to muscle activation, during voluntary isometric contractions, the increase in ventilation has been shown to be proportional to the relative intensity of the central motor command (Myhre and Andersen 1971; Muza et al. 1983; Imms and Mehta 1989) rather than the size of the activated muscle mass (Imms and Mehta 1989). That is, a small muscle contracting at the same percentage of maximal force output as a larger muscle would produce similar increases in ventilation. This finding was interpreted as central signals providing the dominant input to respiratory premotoneurones during isometric contractions. Muza et al. (1983) further showed that the increase in ventilation during a sustained handgrip contraction had a similar time course to the increase in the perceived effort of the handgrip contraction. Muza et al. viewed this as further validation for a centrally driven ventilatory response since the sense of effort is also believed to arise from a corollary of the central motor command (Sperry 1950; McCloskey 1981). The perception of effort during a voluntary contraction is distinct from peripherally generated sensations of muscle tension and increases proportionally with contraction intensity as well as during muscle fatigue when an increasing motoneuronal drive is required to maintain an absolute submaximal force output (see Enoka and Stuart 1992; for review). However, the relationship between ventilation and contraction intensity has only been determined for motor tasks involving a contraction of a single muscle group, for example, during a handgrip or knee extension. Extrapolating these findings to everyday motor tasks that typically involve more than one contraction is difficult as it is not clear at which point along the central motor pathway that these corollary signals for ventilation are generated. For dynamic exercise, the centrally generated ventilatory response is also believed to arise from corollaries of the central motor command, although the origin of these corollary signals, either from the cortex or supraspinal locomotor centers, is still debated (Eldridge et al. 2006; Haouzi 2006; Secher 2006; Waldrop and Iwamoto 2006).

In this study, we examined the changes in ventilation and perceived effort of contraction during separate and simultaneous isometric contractions of the contralateral elbow flexors and of an ipsilateral elbow flexor and knee extensor. We hypothesized that if the increase in ventilation is proportional to the output of the motor cortex, then increasing the total area of motor cortical activation with two simultaneous contractions would potentially evoke a ventilatory response twice as large as a separate contraction when performed at the same intensities. On the other hand, if the corollary signals for ventilation are generated from higher centers during the formulation of the movement, as is suggested by the increase in ventilation observed in hypnotized subjects performing an imagined cycling exercise (Thornton et al. 2001), then the ventilatory response would be independent from the total area of motor cortical activation. Our experiments were performed during normocapnia and then repeated during hypercapnia for two purposes, to assess the effect of hypercapnia on (1) the magnitude of the initial ventilatory response to isometric muscle contraction as the activity of respiratory premotoneurones is modulated by CO_2_ and (2) the perceived effort of muscle contraction since the larger excursions of the chest wall and abdomen during hypercapnia may have led to an increased sensation of physical exertion.

## Materials and Methods

Eight healthy adults (five female) aged 23–47 years took part in the experiments. Subjects gave their informed consent in writing. All procedures were approved by the Human Research Ethics Committee at the University of New South Wales and conducted in accordance with the Declaration of Helsinki.

### Respiratory measurements

Respiratory variables were recorded continuously as subjects wore a nose clip and breathed room air via a mouthpiece attached to a two‐way valve. A pneumotachometer (Series 3813, Hans Rudolph Inc., Kansas City) on the inspiratory line was connected to a differential pressure transducer (DP45‐16, Validyne Engineering Corp., Northridge) to measure inspiratory flow, which was integrated on‐line to obtain inspired lung volume. Arterial CO_2_ was estimated from end‐tidal CO_2_, as measured close to the mouth piece with a capnometer (Normocap, Datex Instrumentarium Corp., Helsinki, Finland). For conditions that required an elevated end‐tidal CO_2_, a rebreathing circuit consisting of a reservoir bag was connected in series to a T‐piece valve and the two‐way valve. The T‐piece valve was manually operated to titrate room air into the rebreathing circuit to regulate the level of end‐tidal CO_2_. Oxygen saturation was monitored continuously with a pulse oximeter (Biox 3740, Ohmeda, Louisville) and remained above 95% in all subjects.

### Protocol

#### Experiment 1: Perceived effort and ventilation during separate versus bilateral arm contractions

**Figure 1:**
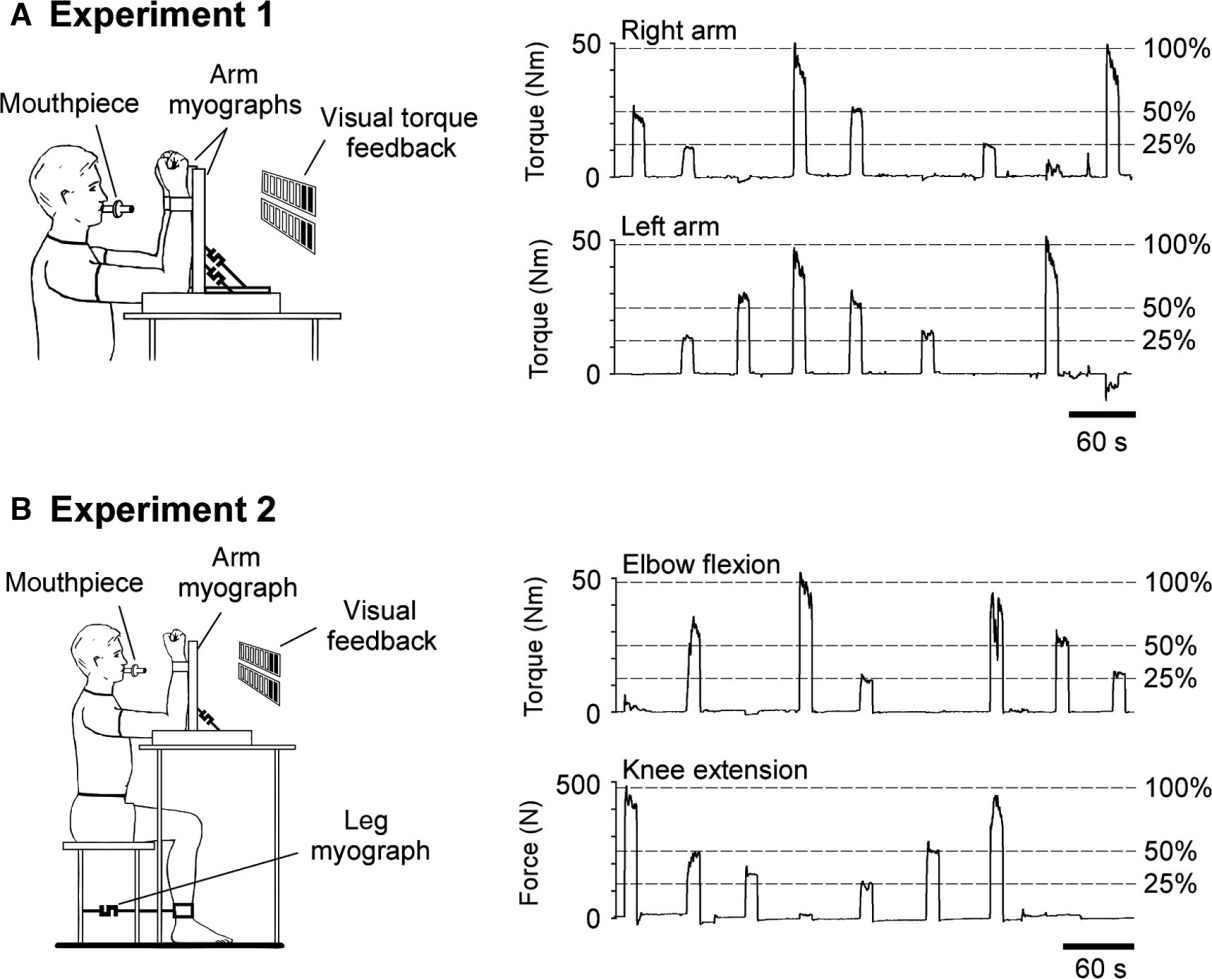
Experimental setup and protocol. Respiratory measurements were recorded continuously in all experiments as subjects breathed through a mouthpiece attached to a pneumotachograph. (A) For separate and bilateral contractions of the elbow flexors, subjects were seated comfortably with both arms placed in separate myographs. The force generated by elbow flexion was recorded from 2 kN load cells (XTRAN, Applied Measurement Australia, Mitcham, Australia), one for each arm. Continuous recordings for a single set of contractions of the right and left elbow flexors are shown in the right panel. Horizontal dashed lines indicate the contraction intensity relative to maximal torque output. (B) Subjects were seated for contractions of the right elbow flexors and right knee extensors. Elbow‐flexor torque was measured using the arm myograph and knee‐extensor force was recorded from a load cell (XTRAN, Applied Measurement Australia) attached to the base of the chair and a strap around the ankle joint. Continuous recordings for a single set of elbow‐flexion and knee‐extension contractions are shown in the right panel

Seven subjects participated in Experiment 1. Subjects sat comfortably with both arms flexed at 90°and placed in separate myographs that measured flexion torque at the elbow (Fig. 1A). Both forearms were held vertical and supinated in the myographs by straps just proximal to the wrist. Visual feedback of each elbow's flexor torque was provided to the subject on separate panels of light‐emitting diodes.

Prior to contractions of the elbow flexors, a short period of quiet breathing was recorded for each subject to determine the baseline ventilation and end‐tidal CO_2_ at rest. Subjects then maximally flexed the right and left elbows separately to determine their maximal elbow‐flexor torques. To compare the perceived effort and ventilation of separate versus bilateral arm contractions, subjects were instructed to perform two sets of isometric contractions of the elbow flexors. Each set required subjects to briefly contract the left and right arms separately and both arms simultaneously at the same contraction intensities to target torques of 25, 50, and 100% maximum (Fig. 1A). These contractions were performed in random order and at normocapnia. Contractions were sustained for 10 sec with a minimum 30‐sec rest period between contractions to avoid muscle fatigue. Subjects were instructed to continue breathing during the contractions to avoid excessive bracing of the body, particularly during maximal isometric contractions where ventilation can be disrupted (Dejours 1964). During the rest period, subjects reported their perceived effort of elbow flexion by signaling a number with their fingers. They could not give a verbal rating as they were breathing through a mouth piece. While no upper limit was set on ratings of effort for the range of contractions because we did not wish the participants to be limited to a perceived “maximal” rating that could not be exceeded, subjects were advised to use the same criteria to rate each contraction and that it would be difficult to signal large numbers with their fingers. However, ratings higher than 10 could be indicated by signaling 10 fingers, then extra digits after that. Half‐integer ratings were permitted by signaling with a bent index finger immediately after reporting the desired integer. Using the same protocol outlined here, two additional sets of contractions were repeated at hypercapnia to reflexly increase baseline ventilation. Rebreathing via an added reservoir bag was employed to produce a twofold increase in ventilation. This resulted in an elevated end‐tidal CO_2_ from 36.5 ± 3.0 mmHg (mean ± SD) to 47.9 ± 3.8 mmHg at rest.

#### Experiment 2: Perceived effort and ventilation during ipsilateral arm and leg contractions

Eight subjects participated in Experiment 2; seven of whom had completed Experiment 1. Using a similar protocol to Experiment 1, the setup was modified to compare the ventilation and perceived effort of contraction of the elbow flexors to a contraction of the knee extensors when performed separately and simultaneously. Subjects were seated with the right forearm placed in a myograph while the left arm was relaxed by the side (Fig. 1B). With the knees flexed at 90°, the right ankle was secured to a myograph to record knee extensor forces. Subjects performed isometric contractions of the right arm and leg separately and simultaneously at 25, 50, and 100% of the predetermined maximums for each limb. Before each knee extension, subjects were instructed to take up the slack of the ankle strap. Two sets of contractions were made at normocapnia and a further two sets were performed at hypercapnia.

### Data extraction

Data were acquired (Power 1401, Cambridge Electronic Design, Cambridge, UK) and recorded to computer with Spike2 software (Cambridge Electronic Design). Respiratory data were sampled at 1000 Hz. Torque and force were sampled at 2000 Hz.

Ventilation and end‐tidal CO_2_ were computed over three breaths prior to and during each 10‐sec contraction, and then averaged for each contraction condition. For one subject during the single‐arm contractions at 25% maximum, ventilation and end‐tidal CO_2_ were averaged over two breaths due to a slightly reduced breathing frequency. Ventilation was then normalized as a percentage of the baseline ventilation at rest during normocapnia.

Ratings of perceived effort of contraction for upper and lower limb muscles were normalized for each subject to compare across conditions. The effort of contraction reported during maximal activation of the right elbow flexors at normocapnia was scaled to produce a rating of 10, with the same scaling factor applied to all of the subject's other ratings. As there was no difference between effort ratings for separate contractions of the left and right arms in Experiment 1, the normalized perceived effort of contraction for each arm was averaged to produce one single‐arm rating at each contraction intensity.

### Statistical analyses

For Experiment 1, the perceived efforts of the elbow flexors during normocapnia were analyzed in a two‐way repeated measures analysis of variance (ANOVA) with factors Arms (separate vs. bilateral) and Contraction Intensity (25, 50, and 100% maximum). Ventilation during normocapnia was analyzed in a separate two‐way repeated measures ANOVA with Arms and Contraction Intensity as factors, with baseline ventilation at rest included as an additional level for Contraction Intensity. To determine the effect of hypercapnia on the contraction‐induced ventilatory response, the difference in ventilation between separate and bilateral arm contractions at the different levels of end‐tidal CO_2_ was compared in a two‐way repeated measures ANOVA with CO_2_ (hypercapnia vs. normocapnia) and Contraction Intensity as factors. The effect of hypercapnia on perceived effort was analyzed using a similar design to compare the difference in perceived effort between separate and bilateral arm contraction during normocapnia with hypercapnia.

For Experiment 2, the perceived efforts of limb contractions during normocapnia were analyzed in a two‐way repeated‐measures ANOVA with factors Limb (arm, leg, and simultaneous) and Contraction Intensity (25, 50, and 100% maximum). Ventilation during normocapnia was analyzed with a similar ANOVA design with baseline ventilation at rest included as an additional group for Contraction Intensity. To determine the effect of hypercapnia on the contraction‐induced ventilatory response, the difference in ventilation between knee extension and elbow flexion at the different levels of end‐tidal CO_2_ was compared in a two‐way repeated measures ANOVA with CO_2_ (hypercapnia vs. normocapnia) and Contraction Intensity as factors, and the difference in ventilation between simultaneous contractions and elbow flexion alone was assessed in a separate two‐way repeated measures ANOVA with the same design. The difference in perceived effort between knee extension and elbow flexion at the different levels of CO_2_ was compared in a two‐way repeated measures ANOVA with CO_2_ (hypercapnia vs. normocapnia) and Contraction Intensity as factors. The difference in perceived effort between simultaneous contractions and elbow flexion alone was assessed in a separate two‐way repeated measures ANOVA with the same design as for knee extension. Where post hoc comparisons were required, Bonferroni corrected t‐tests were used. Group mean data are presented as means ± standard deviations (sd).

## Results

### Experiment 1: Separate versus bilateral arm contractions

**Figure 2:**
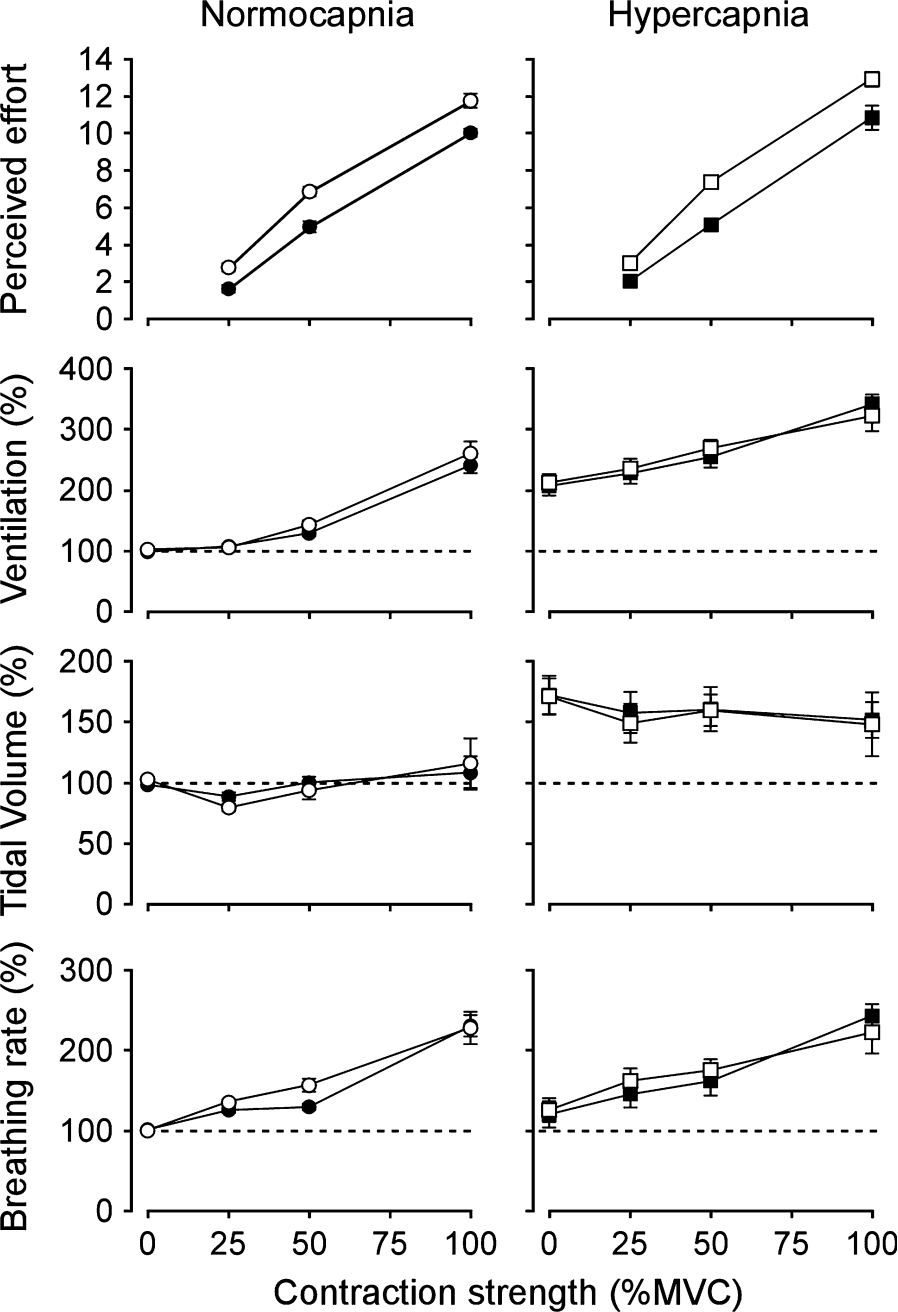
Separate versus bilateral arm contractions. Normalized group mean data are shown for the perceived effort of contraction of separate (filled symbols) and bilateral (open symbols) contractions of the elbow flexors during normocapnia and hypercapnia. Ventilatory data were measured at rest and during muscle contractions. Data recorded during left arm‐only or right arm‐only contractions have been averaged and are presented as the single‐arm contraction condition. Both the perceived effort of contraction and ventilation increased with contraction strength (%MVC, maximal voluntary contraction) during separate and bilateral contractions of the elbow flexors. The increase in ventilation during hypercapnia did not affect the perceived effort of contraction of the elbow flexors. Data are presented as means ± SD. Horizontal dashed lines represent the normocapnic baseline at rest.

As expected, subjects perceived stronger contractions to be more effortful (*F*_2,12_ = 404.5, *P* < 0.001, Fig. 2, left panel) and with increasing contraction intensity, ventilation also increased (*F*_3,18_ = 59.5, *P* < 0.001). The mean effort rating for a single‐arm contraction of the elbow flexors during normocapnia at 25% MVC was 1.5 ± 0.5, which increased to 4.8 ± 0.8 at 50% MVC and 9.9 ± 0.5 at 100% MVC. These single‐arm contractions produced increases in ventilation with increasing contraction intensity, from 16.3 ± 3.9 L min^−1^ at rest to 39.4 ± 8.6 L min^−1^ at 100% MVC. This contraction‐induced increase in ventilation was achieved through increases in breathing frequency rather than tidal volume (Fig. 2, left panel). When subjects contracted both arms simultaneously, they did not report that bilateral‐arm contractions required twice as much effort as a single‐arm contraction. There was, however, a small but significant increase in the perceived effort of contraction for bilateral compared to single‐arm contractions (*F*_1,6_ = 71.7, *P* < 0.001). Bilateral‐arm contractions at 25% MVC were rated as 2.6 ± 0.6, at 50% MVC they were rated as 6.8 ± 0.6, and at 100% MVC the perceived effort was rated as 11.6 ± 1.0 (a 17.4% increase compared to single‐arm contractions). Ventilation during bilateral‐arm contractions also increased with contraction intensity during normocapnia, from 16.8 ± 4.1 L min^−1^ at rest to 42.3 ± 9.5 L min^−1^ at 100% MVC; but this was not significantly different from a single‐arm contraction (*F*_1,6_ = 3.8, *P* = 0.1).

When these contractions were repeated at hypercapnia to increase the feedback of muscle and chest wall afferents, baseline ventilation increased twofold to 35.5 ± 11.9 L min^−1^ at rest. This hypercapnia‐induced increase in ventilation was mainly due to changes in tidal volume rather than breathing frequency (Fig. 2, right panel). Ventilation increased further with contraction strength, increasing to 56.8 ± 11.1 L min^−1^ for a single‐arm contraction at 100% MVC and to 52.7 ± 11.2 L min^−1^ for bilateral‐arm contractions at 100% MVC. Similar to normocapnia, the contraction‐induced increase in ventilation was achieved mainly through an increase in breathing frequency. Despite the increase in baseline ventilation during hypercapnia, the difference in ventilation at each contraction intensity between the bilateral and single‐arm contractions was not significantly different when contractions were performed at normocapnia and hypercapnia (*F*_1,6_ = 1.8, *P* = 0.22). These findings suggest an additive effect of contraction‐induced increases in ventilation on top of the hypercapnia‐induced increase. There was also no significant effect of hypercapnia on the difference in perceived effort of contraction between bilateral and single‐arm contractions when compared to normocapnia (*F*_1,6_ = 0.86, *P* = 0.39).

### Experiment 2: Ipsilateral arm and leg contractions

**Figure 3:**
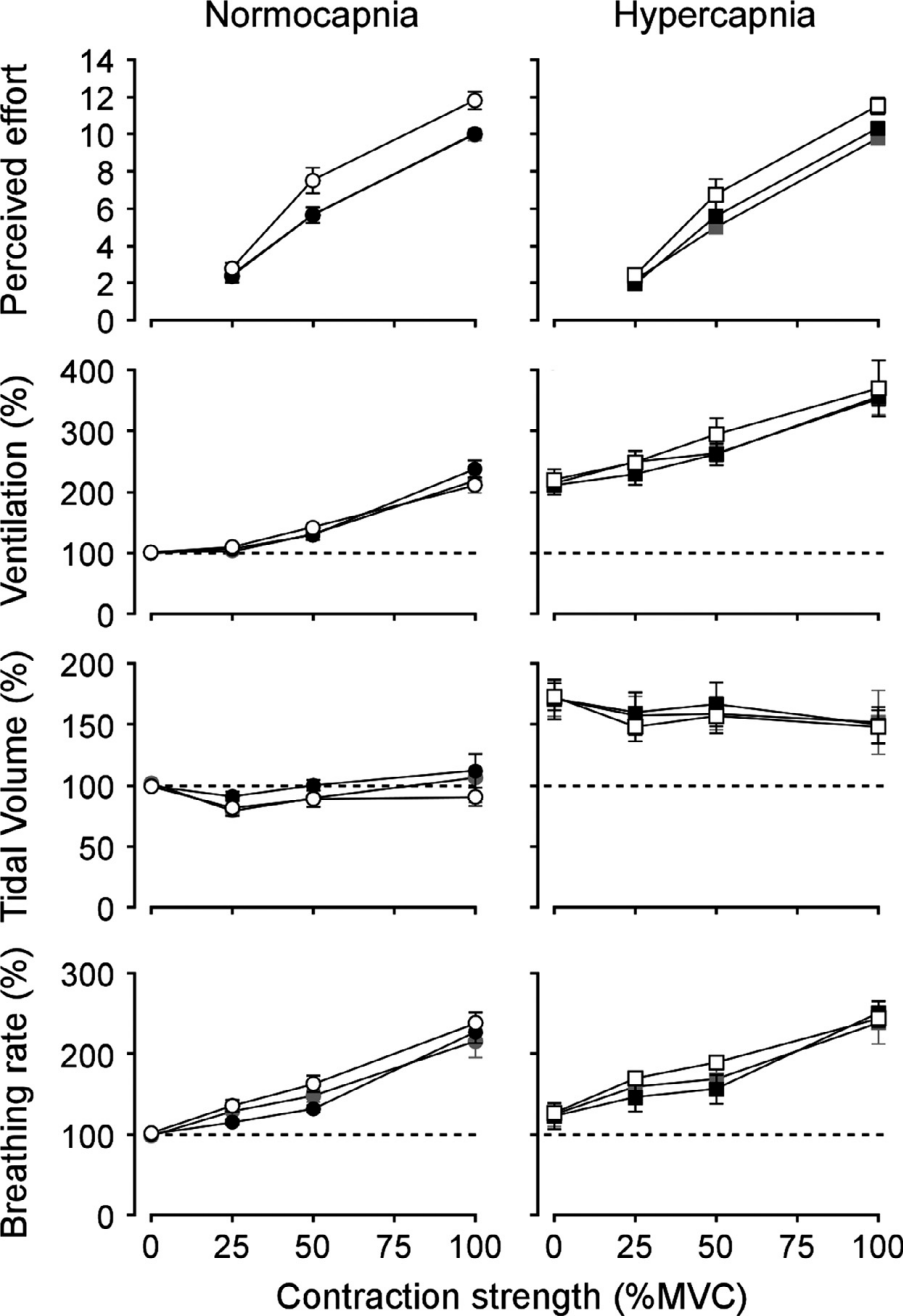
Arm and leg contractions. Normalized group mean data are shown for contractions of the right elbow flexors (black symbols), right knee extensors (grey symbols), and elbow flexors and knee extensors simultaneously (white symbols). During normocapnia, the perceived efforts of contraction of the knee extensors were similar to the elbow flexors across contraction strength (%MVC, maximal voluntary contraction), and as a result, the data points for the knee extensors are obscured by the elbow flexors. Ventilatory data were recorded at rest and during muscle contractions, with hypercapnia approximately doubling ventilation at rest. Data are presented as means ± SD. Horizontal dashed lines represent the normocapnic baseline at rest.

When contractions were performed using different muscle groups but from the motor cortex of the same hemisphere, subjects produced similar results to Experiment 1. Ratings of perceived effort increased with contraction strength (*F*_2,14_ = 263.9, *P* <0.001) and differed across limb conditions (*F*_2,14_ = 13.8, *P* <0.001). Ratings for the perceived effort of knee‐extension contractions were not significantly different from ratings of elbow flexion at the same contraction intensities (*P* = 1.0, Fig. 3). Similar to the bilateral‐arm contractions, subjects did not report that simultaneous elbow flexion and knee extension at the same contraction intensities required twice as much effort as single limb contractions, but there were small significant increases from both elbow flexion alone (*P* = 0.003) and knee extension alone (*P* = 0.02, Fig. 3). For example, with simultaneous contractions compared to elbow flexion alone, ratings of perceived effort increased by 0.4 ± 1.1 at 25% MVC, 1.9 ± 1.2 at 50% MVC, and 1.8 ± 1.3 at 100% MVC (an increase of 17.8%). The increase in ventilation with increasing muscle contraction intensity (*F*_3,21_ = 48.2, *P* < 0.001) was not significantly different for separate or simultaneous contractions of the elbow flexors and knee extensors (*F*_2,14_ = 0.958, *P* = 0.407). Repeating these contractions during hypercapnia did not affect the perceived effort of contraction of the elbow flexors and knee extensors. The difference in perceived effort of contraction between knee extension and elbow flexion during normocapnia was not significantly changed during hypercapnia (*F*_1,7_ = 1.7, *P* = 0.238), nor was the difference in perceived effort between simultaneous contractions of these two muscle groups and elbow flexion alone (*F*_1,7_ = 2.9, *P* = 0.13). The effect of hypercapnia on the contraction‐induced ventilatory response was mixed for separate and simultaneous contractions. Overall, the mean difference in ventilation between knee extension and elbow flexion was greater during hypercapnia than normocapnia; but this increase of 10.2 ± 16.3% of baseline ventilation at rest was not significant (*F*_1,7_ = 2.2, *P* = 0.182). During simultaneous contractions of the knee extensors and elbow flexors, however, the mean difference in ventilation compared to elbow flexion alone was significantly greater during hypercapnia than normocapnia, an increase of 20.6 ± 18.7% of baseline ventilation at rest (*F*_1,7_ = 6.8, *P* = 0.035).

## Discussion

These experiments investigated the ventilatory response and perceived effort of contraction at the onset of muscle activity. Our results show that the ventilatory response produced during isometric muscle contractions of a single muscle group scales with contraction intensity, as described previously (Myhre and Andersen 1971; Muza et al. 1983; Imms and Mehta 1989), but does not show equivalent increases when two muscle groups are activated simultaneously at the same relative intensities. Additionally, subjects did not perceive two simultaneous muscle contractions as requiring twice as much effort as a contraction of a single group at the same intensity and effort did not reflect the combined muscle force. These findings were consistent regardless of whether the simultaneous muscle contractions were performed with contralateral or ipsilateral muscle groups, which suggests that the ventilatory response and perceived effort of contraction at the onset of muscle activation are not related to the total output of the primary motor cortices. The results also show that hypercapnia did not increase the perceived effort of contraction of the limb muscles, and that the ventilatory response to muscle contraction is separate from but summates with the hypercapnia‐induced ventilatory response since contraction‐induced increases in ventilation were achieved through changes in breathing frequency rather than tidal volume (Smith et al. 2008).

### Ventilation

A key finding in this study is that when two muscle groups are contracted simultaneously at the same relative intensity, the increase in ventilation is not equal to the sum of the individual ventilatory responses for each muscle group. Although ventilation was proportional to contraction intensity for simultaneous contractions, the increase in ventilation resembled the ventilatory response for only a single muscle group. This was true whether the concomitant contractions involved contralateral muscle groups of similar size and absolute tension or ipsilateral muscle groups with large differences in size and absolute tension. This finding indicates that at the onset of voluntary isometric contractions, any peripheral input from the contracting muscles to the ventilatory response, including feedback from group III and IV muscle afferents (see Amann et al. 2010), would be small, and supports previous interpretations that central corollary signals provide the dominant input to respiratory premotoneurones (Myhre and Andersen 1971; Muza et al. 1983; Imms and Mehta 1989). The origin of these central contributions to the ventilatory response has not yet been identified. Our findings rule out any direct contributions from the primary motor cortices down to the neuromuscular junction, since increases in ventilation were not proportional to the combined output of the motor pathways for the voluntary limb muscle contractions. During voluntary limb muscle contractions, postural muscles are also recruited to varying extents for stabilization, so that it is an oversimplification to state that the two‐limbed contractions involve exactly “double” the motor output or muscle force. However, the two‐limbed contractions will significantly increase the total force output in comparison to the single‐limb contraction and activity in the motor cortex would also be expected to increase substantially. For bilateral maximal efforts of the index finger, functional imaging shows strong activation of primary motor cortex bilaterally in contrast to the largely contralateral activation with unilateral efforts (Post et al. 2007). Nevertheless, the total cortical activation is less than exactly twice that of two separate contractions (Post et al. 2007), possibly through transcortical inhibition (Wu et al. 2008; Vidal et al. 2014). As the bilateral deficit is small, this does not explain why ventilation does not increase at all when additional muscle groups are activated. Indirect contributions from spinal locomotor circuits (Haouzi 2006; Waldrop and Iwamoto 2006) can also be excluded since these experiments involved isometric contractions with the subject seated. It seems most likely then that higher brain centers are involved in setting the initial ventilatory response to isometric muscle contraction. Functional imaging studies on hypnotized subjects performing imagined cycling have implicated cortical regions involved in motor planning, the dorsolateral prefrontal cortex, supplementary motor areas and premotor areas (Thornton et al. 2001), which may also apply to isometric muscle contractions.

The nonproportional nature of the central ventilatory response in relation to concomitant muscle contractions is not unique to the respiratory system. Lind and McNicol (1967) described a similar behavior for the cardiovascular responses during separate and simultaneous muscle contractions. They showed that when two or more muscle groups were contracted isometrically at the same intensity, the increases in heart rate and blood pressure were the same as when only a single muscle group was contracted. Lind and McNicol further showed that for simultaneous muscle contractions at different relative intensities, the cardiovascular response was the same as when the muscle group at the higher relative tension was contracted separately. It is important to note that Lind and McNicol's study described the cardiovascular responses toward the end of sustained contractions rather than at the onset, and therefore, may be susceptible to greater peripheral influences than observed in this study. Any effect, however, would likely be small since increasing the total muscle mass activated through simultaneous contractions did not produce a larger increase in the cardiovascular response than when contracting a single muscle group alone. The similarity between the respiratory and cardiovascular responses to voluntary isometric contractions is not surprising given their respective roles in supplying O_2_ for muscle metabolism and in maintaining normocapnia. Moreover, the cardiovascular response is also scaled relative to the intensity of the central motor command rather than the absolute force produced by the muscle (Goodwin et al. 1972; Leonard et al. 1985). One possible advantage of having the ventilatory response independent from the total output of the motor pathways is that it negates the need for a large excess capacity. Certainly, during normocapnia, the ventilatory responses for maximal limb contractions did not approach the limit of voluntary ventilation reported by Smith et al. (2008). Even with the hypercapnia‐induced increase in baseline ventilation at rest, ventilation during maximal limb contractions was only ~50% of the voluntary maximum (c.f. Smith et al. 2008). In contrast, if ventilation scaled with the number of muscle groups, the simultaneous activation of more than two muscle groups could potentially drive ventilation toward maximum at the onset of only moderate levels of muscle activity. Further investigations are required to understand the functional consequences of a ventilatory response that scales with contraction intensity but not to the total output of the motor pathways.

Our results also showed that the centrally driven ventilatory response to muscle contractions is separate from a CO_2_‐driven change in ventilation. Previous studies involving dynamic exercise have suggested an interaction between chemical and neural stimuli to respiratory premotoneurones as hypocapnia prior to the onset of exercise delayed the initial ventilatory response (Asmussen 1973; Ward et al. 1983). In this study, we investigated the effect of an increased respiratory drive on the initial ventilatory response to muscle contraction by inducing hypercapnia prior to muscle activity. Hypercapnia increased baseline ventilation as expected, but did not affect the ventilatory response to separate or simultaneous limb contractions. Rather the ventilatory responses to chemical and neural stimuli were additive and operated via different mechanisms. The hypercapnia‐induced shift in baseline ventilation at rest was achieved primarily through an increase in tidal volume (Rebuck et al. 1974), whereas the contraction‐induced increase in ventilation resulted from a faster breathing rate (Figs. 2, 3). This discrete increase in respiratory rate with muscle activation implicates the lateral regions of the parabrachial and the Kölliker‐Fuse nuclei, which have been shown to modulate the respiratory rate in animals (Lara et al. 1994) and mediate inspiratory–expiratory phase transitions as well as the coupling of respiratory and cardiovascular activity (Dutschmann and Dick 2012). The lack of modulation of the ventilatory response at the onset of muscle contraction by hypercapnia is consistent with a previous finding that involved sustained isometric handgrip contractions for 5 min. Muza et al. (1983) showed that the ventilatory response during single handgrip contractions was the same whether end‐tidal CO_2_ decreased due to contraction‐induced hyperventilation, or whether CO_2_ was maintained. In their study, Muza et al. created an apparent hypercapnia by preventing the normal decrease in end‐tidal CO_2_, whereas our study shows a similar result with a real increase in end‐tidal CO_2_ and extends this finding to simultaneous contractions.

### Sense of effort

Like the central ventilatory response to muscle contractions, the perceived effort of a voluntary contraction is considered to be derived from a corollary of the central motor command and reflects the intensity of the motor drive (Sperry 1950; McCloskey 1981). Here, we have shown that the perceived effort of contraction had a similar pattern of behavior to the ventilatory response. Subjects reported that the perceived effort of contraction increased with contraction intensity. However, contracting two muscle groups simultaneously at the same intensity did not feel twice as hard as when either of the muscle groups were contracted separately. The same arguments used for the ventilatory response to rule out any direct contributions from the primary motor cortices down to the muscles also apply here. Primarily, the perceived effort of contraction did not scale with the total output of the motor pathways. This finding is consistent with the report by Gandevia et al. (1993) that the muscle response evoked with transcranial magnetic stimulation of the motor cortex is not accompanied by any sensation of effort. Based on these findings, we conclude that the corollary signals that give rise to the sense of effort originate from higher cortical regions.

Although effort intensity did not reflect the combined muscle output during simultaneous contractions, there was a small increase in the perceived effort compared to individual muscle contractions that was not present in the ventilatory responses. There are three possibilities that may explain the discrepancy between these two corollary derived behaviors. First, the sense of effort is a subjective measure, and therefore, is more susceptible to conscious influences than the ventilatory response to muscle contraction. An increase in cognitive load when maintaining two contractions simultaneously at a fixed intensity opposed to a single contraction may have been interpreted as an increase in the effort of contraction. Second, despite explicit instructions to report the effort perceived during the contractions, it is possible that subjects may have been biased by peripheral sensations of force or tension developed within the contracting limb muscles (McCloskey et al. 1974). This seems unlikely; the combined absolute muscle tension developed during ipsilateral contractions of the elbow flexors and knee extensors was much larger than bilateral contractions of the elbow flexors, but there was no difference in the perceived effort of these contractions relative to the right elbow flexors, an increase of 17.8 and 17.4%, respectively, at 100% MVC. The third possibility is that subjects were guided by how hard they were breathing during the limb contractions and reported physical exertion rather than the effort of contraction. However, in normocapnia, the ventilatory responses were similar for simultaneous and separate contractions and presumably any respiratory‐related sensations from afferents in the contracting respiratory muscles, the upper airway, and the chest wall were also similar.

The effect of an increased sensation of breathing on the perceived effort of a limb contraction was investigated further using hypercapnia to automatically increase baseline ventilation. As reported earlier, hypercapnia doubled ventilation at rest and summated with the ventilatory response to muscle activation to produce even larger respiratory movements of the chest wall; but this did not affect the perceived effort of a limb contraction. The difference in perceived effort between simultaneous and separate limb contractions during normocapnia did not change with hypercapnia. This result shows that subjects were able to distinguish between the effort of a voluntary limb contraction and the respiratory‐related sensations associated with physical exertion. Additionally, the lack of an effect by hypercapnia on the perceived effort of a limb contraction argues against any direct contributions from medullary respiratory centers to the sense of effort and provides further evidence that the neural signals that give rise to this sensation originate upstream from the primary motor cortex.

**Figure 4:**
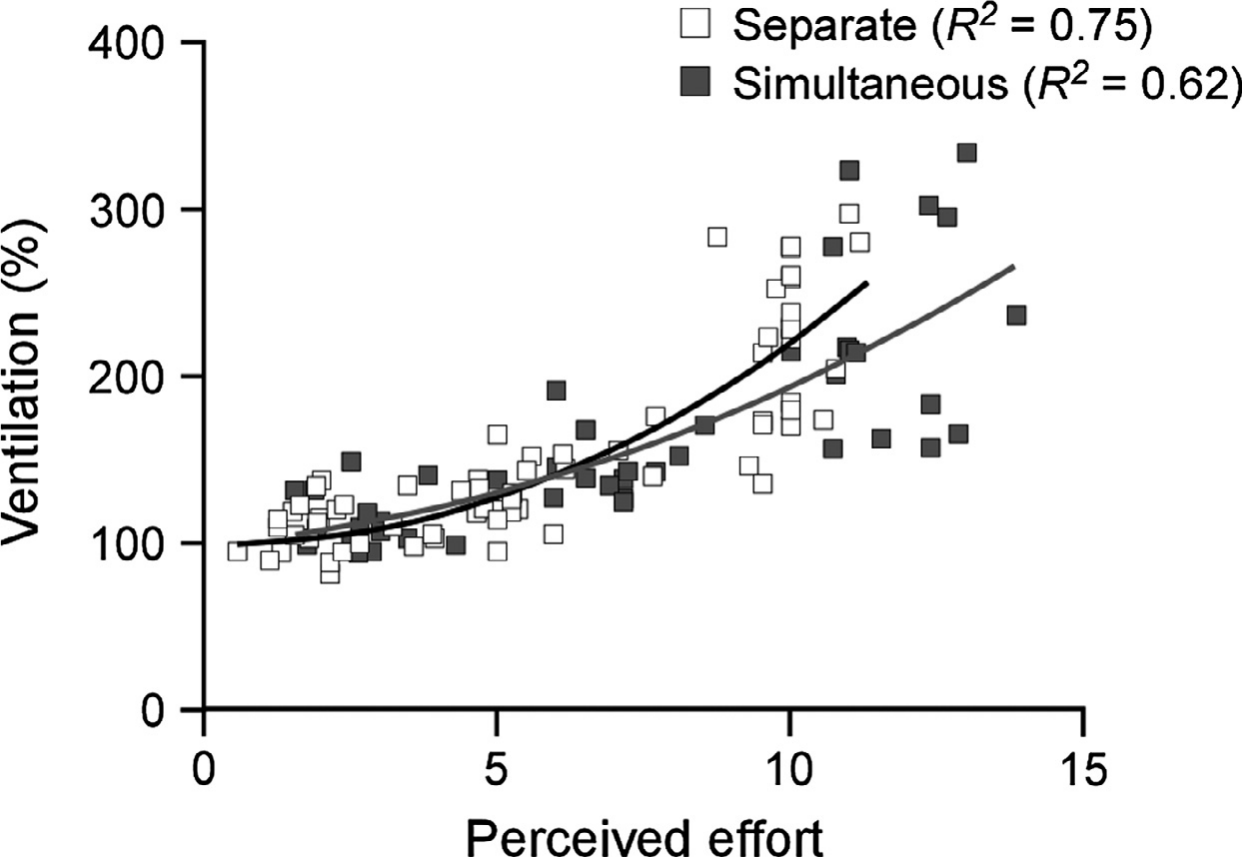
Relationship between ventilation and effort. Normalized data from all subjects for ventilation and perceived effort are shown for each muscle contraction during normocapnia. Data for separate contractions of the elbow flexors and knee extensors were best fitted by a quadratic function (1.3214x^2^ − 1.2276x + 100) with the y‐intercept set at 100%, the normalized ventilation at rest. Data for simultaneous contractions of the contralateral elbow flexors and of the right elbow flexors and knee extensors were described by the function 0.6884x^2^ + 2.5267x + 100.

A correlation between the ventilatory response and sensation of effort has been referred to previously by Muza et al. (1983) for a fatiguing handgrip contraction in a single limb. They indicated that the time course for the increase in perceived effort during a fatiguing contraction closely resembled the time course for the slow rise in ventilation, and the peak magnitudes for perceived effort and ventilation were both dependent on contraction intensity. Here, we report a similar finding at the onset of an isometric contraction with the magnitudes for both perceived effort and the ventilatory response increasing proportionally with contraction intensity. Figure 4 shows that the relationships between ventilation and effort for separate and simultaneous contractions are best described by quadratic functions. At low to moderate contraction intensities, the regression lines are comparable for separate and simultaneous contractions, which is consistent with them being driven by central signals upstream from the primary motor cortex. It is only during maximal contractions that the predicted behaviors begin to diverge. Whether this represents a true difference in neural mechanisms when contracting at 100% MVC is not clear as there was increased variability in both ventilation and effort during maximal contractions. Nevertheless, these correlations between ventilation and effort, in conjunction with the cardiovascular response to sustained contractions (Lind and McNicol 1967), suggest a common origin for these feedforward consequences of isometric contractions and may reflect a general pattern of behavior for neural processes that result from corollaries of the central motor command.

## Conclusions

The main finding in this study was that when subjects performed simultaneous isometric contractions of two muscle groups, the rapid increase in ventilation at the onset of muscle activity and the accompanying sensation of effort were not twice as large as when each muscle group was contracted separately and did not reflect the combined muscle force. The ventilatory response was not significantly different for separate or simultaneous contractions and thus was independent from the total area of motor cortical activation. This study also showed that the ventilatory response to isometric muscle contraction was not affected by and is separate from a hypercapnia‐driven increase in ventilation. The perceived effort of a limb contraction was similarly not affected by the larger breaths produced by hypercapnia. These findings suggest that the central signals that generate the initial ventilatory response and sensation of effort originate from higher areas of the brain than the primary motor cortex.

## Conflict of Interest

None declared.
